# Randomized controlled trial of a pilot PrEP linkage intervention for individuals leaving incarceration in a southern state: Design and baseline characteristics

**DOI:** 10.1371/journal.pone.0312667

**Published:** 2024-12-02

**Authors:** Heather Horton, S. Alexandra Marshall, Mofan Gu, Brooklyn Tody, Timikia Jackson, Nick Zaller

**Affiliations:** Department of Health Behavior and Health Education, College of Public Health, University of Arkansas for Medical Sciences, Little Rock, Arkansas, United States of America; Beth Israel Deaconess Medical Center/Harvard Medical School, UNITED STATES OF AMERICA

## Abstract

**Objective:**

We report baseline characteristics of a pilot intervention, PrEP-Link, which uses a community health worker (CHW) model to provide navigation to PrEP, the daily HIV preventative medication, and other medical and social services upon release from incarceration.

**Trial design and methods:**

This pilot study uses a randomized controlled trial design. The control group receives enhanced standard of care, and the intervention receives enhanced standard of care plus personalized navigation services from the CHW for up to one year. PrEP-Link is modeled after the Transitions Clinic Program, where a CHW who has lived experience of incarceration builds close relationships with individuals and community partners. After COVID-19 restrictions prevented recruitment within local jails, recruitment for this study took place in local reentry centers which house individuals as they near completion of their jail or prison sentence. Data are collected at baseline, 6 months, and 12 months. Planning and reporting was guided by the CONSORT 2010 checklist.

**Results:**

We enrolled 80 participants between September 2021 and April 2023. Thirty-nine participants were assigned to the control group and forty-one to the intervention group. Data collected at baseline included measures of PrEP usage, HIV risk, drug and alcohol use, healthcare usage, and history of incarceration. Analysis of baseline characteristics show comparability of both demographic and HIV-related risk factors between the two arms.

**Conclusions:**

At baseline, participants had high clinical indication for potential PrEP benefit, particularly with respect to self-reported intravenous drug use and condomless sex. Participants responded with high PrEP acceptability within both study arms. Participants reported low preventative healthcare utilization at baseline. Barriers to accessing PrEP among the study population are significant. Results of this pilot RTC will help inform CHW lead PrEP-linkage interventions for people leaving incarceration.

## Introduction

Individuals living in the southern US are disproportionately impacted by incarceration and HIV [1]. As of 2024, states with the highest rates of incarceration (Mississippi, Louisiana, Arkansas, Oklahoma, Texas, Georgia, Alabama and Florida) are all located in the southern US [2]. Additionally, in 2021, southern states accounted for more than half (52%) of the 32,100 estimated new HIV infections among people aged 13 or older [3]. Individuals involved in the criminal-legal (CL) system are at high risk for HIV infection after their release from incarceration because of social and behavioral factors such as housing instability, poverty, mental illness, and substance use disorders [4].

The overlap between incarceration and HIV has disproportionately impacted communities of color, particularly African American/ Black people. In 2020, African American/Black people were 7.8 times more likely to be diagnosed with HIV infection, as compared to white people [5]; and African American/Black males and females have rates of HIV infection 8.1 times and 15 times the rates of their white counterparts, respectively [5]. Relatedly, rates of incarceration among African American/Black people is approximately 5 times greater than that of white people [6].

Pre-exposure prophylaxis (PrEP) is a form of medication that can effectively prevent HIV infection [7]. The efficacy of PrEP has been well established by randomized controlled trials (RCTs) and open label studies [8–12]. The Centers for Disease Control and Prevention (CDC) recommends PrEP for HIV-negative individuals who engage in a wide range of behaviors including IV drug use, sexual relationship with HIV positive partners, and non-monogamous sexual relationships with partners of unknown HIV status [13]. Despite these recommendations, PrEP uptake in real world settings among disadvantaged populations remains low due to lack of knowledge, limited access, and other factors [14, 15]. In Arkansas, where the current study was conducted, in 2020, only about 15% of individuals determined to be high risk of HIV infection were prescribed PrEP [16]. Among individuals involved in the criminal-legal system, PrEP uptake has been especially low. Qualitative data among individuals involved in the criminal-legal system suggest that HIV-related stigma, low knowledge about PrEP and low perceived risk for HIV infection are significant barriers to accessing PrEP in the community [17, 18].

There is an urgent need to focus resources toward individuals involved in the criminal-legal system to prevent morbidity and mortality associated with HIV infection, particularly in the South where the epidemic of mass incarceration is most disparate [19]. However, there are very few evidence-based care-management interventions for people who are at high-risk for contracting HIV after their release from jail.

Community Health Workers (CHWs) are members of the community with specialized training to provide basic health education and assistance with navigating healthcare to members of their community. CHWs specialize in building community partnerships with services relevant to those leaving incarceration, including local reentry centers which house people nearing the end of their prison or jail sentence. A large body of work documents the positive impact of CHWs on patients’ health outcomes [20–22], experiences [23], service utilization, and healthcare costs [21, 24–28]. Prevention-based CHW models to improve health care for criminal-legal involved people potentially yield tremendous benefits to public health including reduced transmission of infectious diseases, decreased substance misuse, improved management of mental illness and chronic medical conditions, lower short- and long-term health costs, less family and community disruption, improved social cohesion, and improved public safety [29].

The Transitions Clinic Network (TCN) has developed a model of incorporating CHWs with a history of incarceration as part of an integrated medical team, and builds close partnerships with local reentry organizations to address social determinants of health [30]. In prior research, the PI partnered with the UAMS Family Medical Center to pilot the Little Rock Transitions program, based on the national TCN model. We found that the use of a CHW was both feasible and acceptable to individuals transitioning from prison to the community in accessing care. This work informed the current study, the goal of which was to develop and implement a CHW-delivered intervention to individuals involved in the criminal-legal system who were at high risk for HIV infection. Here we report baseline characteristics of the population enrolled into the study.

## Methods

We report baseline characteristics for a randomized controlled trial among people recently released from incarceration to pilot test an intervention known as PrEP-Link. PrEP-Link is modeled after the Transitions Clinic Program, and employs a Community Health Worker (CHW) with lived experience of incarceration to provide navigation and linkage services for PrEP and other needs (e.g., medical care and social determinants of health).

### Ethical considerations

This study is conducted in accordance with all applicable government regulations and University of Arkansas for Medical Sciences research policies and procedures. This protocol and any amendments were submitted and approved by the UAMS Institutional Review Board (IRB).

Study staff obtains signed consent from each participant. The informed consent process includes a detailed verbal description of the study by a study staff member. The study staff asks participants a series of questions to ensure that the information contained in the consent form is clearly understood by the participants prior to consenting to participation. Study staff emphasizes that participation is voluntary. Potential subjects participate in an item-by-item reading of the consent with the study staff person.

The consent process takes place in a quiet and private room (either in a small conference room in the jail, Community Corrections and reentry centers, or in a community location), and participants may take as much time as needed to make a decision about their participation. The consent process may also take place over the phone, Zoom, FaceTime, or other platform preferred by the participant to allow for social distancing during the Covid-19 pandemic. Participation privacy is maintained and questions regarding participation are answered. No coercion or undue influence is used in the consent process.

In addition, we have a Certificate of Confidentiality from the National Institutes of Health. The purpose of this certificate is to protect the identity of research subjects participating in studies that collect sensitive information. Because this study collects detailed HIV sexual and substance use behavior among those with criminal-legal system involvement, a Certificate of Confidentiality is necessary.

### Adaptations to the COVID-19 pandemic

We conducted a pilot study of the PrEP-Link intervention at a local county detention center (jail), originally intending to recruit individuals in this same jail for this RCT. However, the COVID-19 pandemic required carceral settings to restrict outside visitors, and we were unable to continue to recruit participants directly from the jail. Therefore, we modified our recruitment protocols to focus on reentry centers, which are carceral settings that house individuals within the final 6–8 months of their jail or prison sentence. Given that reentry centers are short-term facilities, we felt that this change in recruitment strategy was consistent with the original intent of the project to focus on engaging individuals who are at high risk for HIV infection residing in short term carceral settings (see description below).

### Recruitment and enrollment

We recruited participants between September 2, 2021 and April 30, 2023 within local reentry centers, a setting which offered unique access to participants as they transitioned from prison settings back to community life. Reentry centers are privately-run facilities that receive funding through the state of Arkansas to provide housing and other programmatic services for individuals transitioning out of incarceration. Reentry centers may have their own criteria for selecting individuals for their programs, where individuals must follow rules set by the reentry centers or return to incarceration for any remaining portion of their sentence. Individuals in reentry centers are still considered by the state of Arkansas to be incarcerated since their sentence is not yet complete. Reentry centers made an ideal site for study recruitment because it allowed us to reach people nearing the end of their sentence and returning to life outside of incarceration.

Recruitment was initiated through an educational class taught by a CHW and study Research Assistant (RA). The class included general information about HIV and STI prevention, including PrEP. Study staff then provided an overview of the PrEP-Link study. At the end of the class, participants individually filled out a class assessment to provide feedback on the class and discreetly informed study staff if they wanted more information about the study. Study staff contacted participants who indicated they were interested in additional information, and invited them to complete an eligibility screener on site at the reentry center. Study staff gave all class participants a PrEP-Link pamphlet which included information about PrEP medication, payment options, and local HIV and STI testing locations, as well as information about local clinics that prescribes PrEP (including tele-PrEP), and how to make an appointment.

### Eligibility screening

The CHW or study RA performed eligibility screening. Inclusion criteria included: 1) aged 18 or older at study enrollment; 2) able to understand and speak English and to provide written and verbal consent; 3) able to provide at least three reliable pieces of locator information, 4) HIV negative with either an on-site rapid test or self-reported recent (within 12 months) test within a correctional facility; 5) recently released from a correctional facility (within 30 days) and residing in Central Arkansas; 6) Self-reported to have engaged in one or more of the following behaviors that increase risk of HIV infection (per CDC/WHO PrEP guideline) in the last six months:

- Having condomless vaginal or anal sex with more than one partner
- Having a sex partner with one or more HIV risk
- Being diagnosed with a STI
- Reporting history of sharing injection materials/equipment
- Having a sexual partner who is HIV positive

Exclusion criteria included individuals who were unable to provide informed consent, including those with severe mental illness requiring immediate treatment or with mental illness limiting their ability to participate (e.g., dementia).

For those who choose to confirm their HIV status for study inclusion with a rapid test on site, study staff trained by the Arkansas Department of Health administered the FDA-approved OraQUICK ADVANCE Rapid HIV Antibody Tests [31], along with pre- and post-test counseling to interpret test results, increase HIV knowledge, and promote risk reduction. We partnered with Arkansas Department of Health to coordinate confirmatory testing and linkage to care for any reactive tests. We randomized participants to control or intervention arms by using a permuted block technique [32] and separate randomization lists for male and female participants, to capture a representative sample of male participants.

### Control arm

Study staff instructed those assigned to the control arm to contact any of the PrEP providers on the PrEP-Link pamphlet to make an appointment to further assess PrEP eligibility. Study staff advised individuals to call study staff if they had any questions or if they needed any additional information about PrEP or other services in their area. Study staff provided passive referrals as requested. We defined passive referrals as providing information about programs, services, and clinics with PrEP clinicians, but not making appointments, arranging transportation, or performing navigation tasks [33].

### Intervention

In addition to the passive referrals given to all participants, the CHW provided additional support to those in the intervention group. The CHW performed an initial needs assessment with intervention participants to determine immediate needs including social services such as housing, employment, mental healthcare, and primary care. The CHW also offered navigation services in choosing a PrEP provider, making a PrEP appointment, finding transportation, and additional needs related to PrEP linkage.

### Data collection

Data are collected via follow-up meetings or phone calls by research staff using REDCap surveys at baseline, six months, and twelve months after enrollment. Measures include: Criminal-Legal Involvement History (e.g. lifetime incarceration history), Health Insurance (e.g. yes/no and if yes, what type), HIV Sex Risk Perception (e.g. recent sex partners’ HIV risk factors), Income (ASI Lite) [34], PrEP Knowledge (PrEP Knowledge Scale, RADAR) [35], PrEP Attitude [34], PrEP Use [36], PrEP Adherence [34, 37, 38], HIV Attitudes (Perceived Risk of HIV Scale) [39], HIV Risk– 30 days (Texas Christian University HIV/Hepatitis Risk Assessment) [40], Alcohol Use (AUDIT) [41], Substance Use (ASI Lite) [34], Drug Overdose (history of drug overdose), Trauma (Traumatic Life Events Checklist) [42, 43], Depression (Patient Health Questionnaire– 9 item version for Depression Symptoms) [44], Discrimination (Major Experiences of Discrimination Scale) [45], Healthcare Access [46], Health and Utilization [47], Medical Mistrust [48], Social Support (Multidimensional Scale of Perceived Social Support) [49], and Civic Engagement (voter registration status and history).

### Analysis

We extracted baseline data for individuals recruited after September 1, 2021 from REDCap, an electronic data system, and compared participant characteristics in the two trail arms. Ideally, participants in the two trial arms should be similar in demographic profile, HIV risk, PrEP knowledge/history/attitudes, and medical care access, in order to reduce selection bias and confounding. For demographic profiles, we compared sex/gender, race/ethnicity, age, and education attainment. For HIV risk, we compared substance use (heroin, opioids, barbiturates, sedatives, crack/cocaine, amphetamines, cannabis, hallucinogens, and inhalants) within the past 30 days and within lifetime use, injection equipment sharing in the past six months, and condomless sex in the past six months. For medical care access, we compared insurance coverage and type, health care utilization history (time, facility type, number), prescription history (number in the past year), whether the participant had a physical/dental/eye exam in the past 2 years, and whether the participant (female only) had a pap smear in the past 2 years. For statistical testing, we used Chi-Sq Test for categorical variables and Two-Sample t-Ttest for continuous variables. For variables with small cell sizes (<5), we performed Fisher’s Exact Test instead of Chi-Sq Test; for variables with ordinal values (e.g., number of trips to ER/clinic), we performed a Kruskal-Wallis non-parametric Test. P-value greater than 0.05 were considered statistically not significant (in other words, comparable between the two trial arms).

## Baseline results

**Fig 1:**
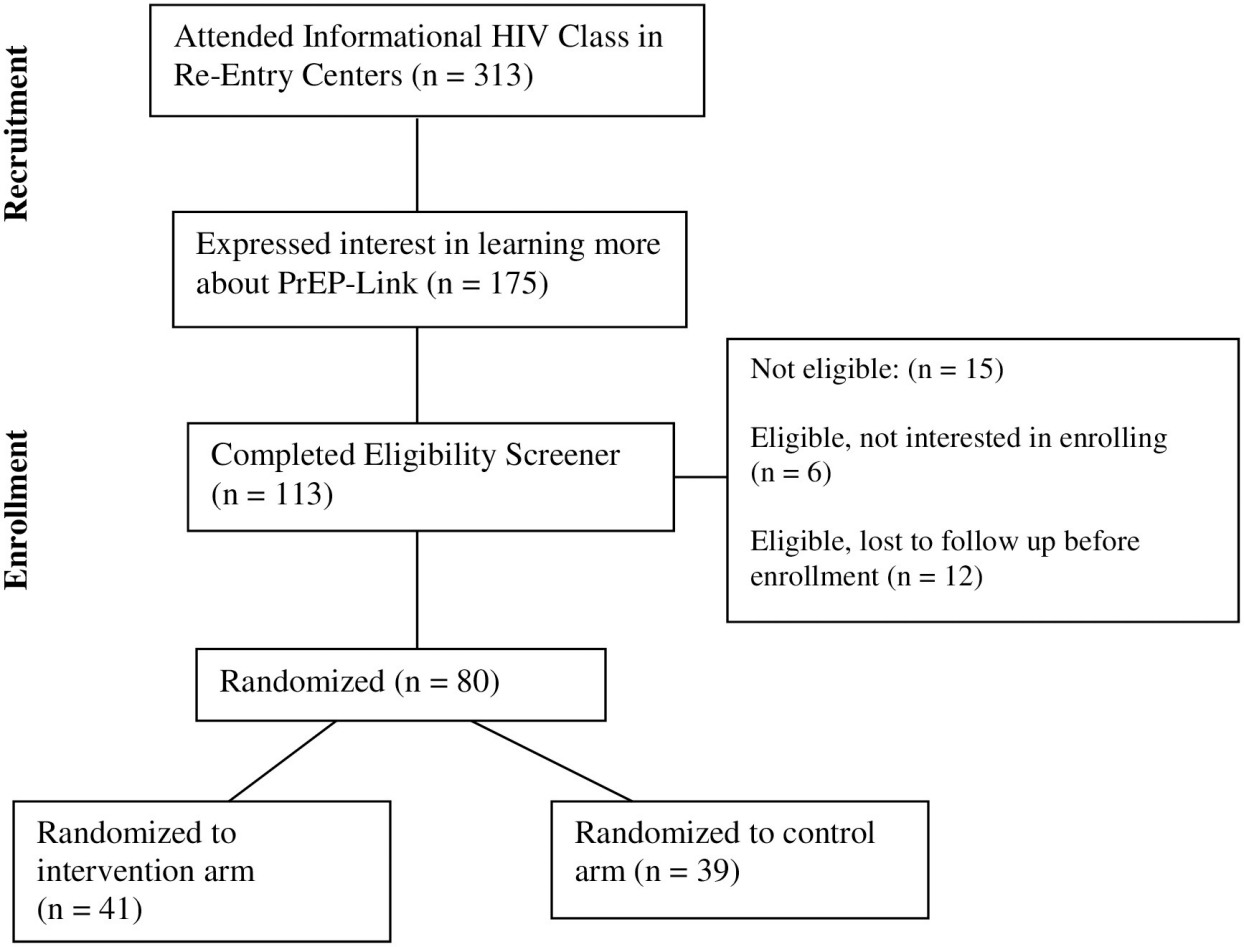
CONSORT participant flow diagram [50].

Study activities for enrolled participants are ongoing, this paper reports on baseline characteristics. The study team enrolled 80 participants between September 2021 and April 2023 at five local re-entry centers in Central Arkansas. A total of 313 individuals in re-entry centers attended a voluntary HIV education class that introduced PrEP as an HIV preventative tool and the PrEP-Link study (Fig 1). Classes received very positive assessments from participants. Of those who attended a class, 175 expressed interest in learning more about PrEP on the class assessment form. One hundred thirteen participants completed an eligibility screening for the PrEP-Link study. Fifteen participants were not eligible due to not meeting HIV risk or location requirements (planning to move outside the study area upon release), six were eligible but not interested in joining the study, and twelve were eligible and initially interested but left the re-entry center prior to enrollment, were re-incarcerated, or were not reachable by study staff to schedule enrollment. Eighty participants were enrolled and randomized.

Baseline measures were collected the same day as study enrollment. The study included 53.8% men and 46.3% women including two trans women. Study participants were 66.3% white and 25% Black/African American with 6.3% of participants identifying as Hispanic. Average age was approximately 37 with the youngest being 18 and the oldest being 63. The majority of participants (67.5%) had a high school education or lower with 7.5% having a college degree (Table 1).

**Table 1:** Participant demographics.

	Control N = 39 (%)	Intervention N = 41 (%)
**Sex Assigned at Birth**		
*Male*	22 (56.4)	23 (56.1)
*Female*	17 (43.6)	18 (43.9)
**Gender**		
*Male*	21 (53.9)	22 (53.7)
*Female*	18 (includes 1 Trans Female) (46.2)	19 (includes 1 Trans Female) (46.3)
**Race**		
*White*	27 (69.2)	26 (63.4)
*Black/African American*	8 (20.5)	12 (29.3)
*Native American*	2 (5.1)	0
*Other*	2 (5.1)	3 (7.3)
**Hispanic Ethnicity**		
**Age**	2 (5.1)	3 (7.3)
*Min*	22	18
*Max*	59	63
*Mean*	36.6	36.7
**Highest Education**		
*8th grade or less*	2 (5.1)	0
*Some high school*	10 (25.6)	7 (17.1)
*GED*	9 (23.1)	11 (26.8)
*High school graduate*	6 (15.4)	9 (22.0)
*Some college*	8 (20.5)	12 (29.2)
*College graduate*	4 (10.3)	2 (4.9)
**Incarceration History**		
*Most recent incarceration (mean*, *months)*	21.84	19.52
*Lifetime incarceration (mean*, *years)*	5.34	5.26

At Baseline, 67.5% percent of participants reported having shared injecting materials or equipment in the 6 months prior to their most recent incarceration. During this same time period, 85% reported having condomless vaginal, oral, or anal sex. Cocaine/crack cocaine was the most commonly reported substance when asked about lifetime use (63.8%), while amphetamines (25%) and cannabis (30%) were most widely reported in the last 30 days prior to the most recent incarceration (Table 2).

**Table 2:** Participant self-reported HIV-related risk factors.

	Control N = 39 (%)	Intervention N = 41 (%)	P-value
**Shared injection materials in 6 months prior to incarceration**	30 (76.9)	24 (58.5)	P = 0.08
**Condomless sex in 6 months prior to incarceration** *	31 (79.5)	37 (90.2)	P = 0.22
**Prior substance use** (“ever” for heroin, opioids, barbiturates, other sedatives, cocaine, amphetamine, cannabis or hallucinogen)	37 (94.9)	36 (87.8)	P = 0.12
**Lifetime substance use:**			
Heroin	12 (30.8)	9 (22)	P = 0.38
Methadone	7 (17.9)	12 (29.3)	P = 0.45
Other Opiates (in a way not prescribed)	15 (38.5)	14 (34.1)	P = 0.90
Barbiturates	12 (30.8)	10 (24.4)	P = 0.54
Sedatives/Hypnotics/Tranquilizers (in a way not prescribed)	16 (41)	13 (31.7)	P = 0.18
Cocaine/Crack Cocaine	26 (66.7)	25 (61)	P = 0.38
Amphetamines	17 (43.6)	21 (51.2)	P = 0.53
Cannabis	21 (53.8)	25 (61)	P = 0.24
Hallucinogens	19 (48.7)	15 (36.6)	P = 0.17
Inhalants	6 (15.4)	8 (19.5)	P = 0.74
**Substance use 30 days prior to incarceration:**			
Heroin*	1 (2.6)	0 (0)	P = 0.51
Methadone*	1 (2.6)	0 (0)	P = 0.51
Other Opiates (in a way not prescribed)	5 (12.8)	5 (12.2)	P = 0.90
Barbiturates*	0 (0)	0 (0)	
Sedatives/Hypnotics/Tranquilizers (in a way not prescribed)*	1 (2.6)	3 (7.3)	P = 0.23
Cocaine/Crack Cocaine*	3 (7.7)	1 (2.4)	P = 0.17
Amphetamines	12 (30.8)	8 (19.5)	P = 0.53
Cannabis	15 (38.5)	9 (22)	P = 0.24
Hallucinogens*	2 (5.1)	0 (0)	P = 0.14
Inhalants*	1 (2.6)	1 (2.4)	P = 0.50

* Fisher’s Exact Test was performed due to small cell numbers in 2*2 tables.

Chi-Squared Test was performed for other variables in the table.

With respect to PrEP use, only one participant had taken PrEP previously before joining the study. Although participants described having little awareness about PrEP prior to the study recruitment and education class, participants who enrolled reported high PrEP acceptability in baseline assessment. The vast majority of participants reported willingness to take PrEP to prevent getting HIV (93.8%) and willingness to take a pill every day to prevent getting HIV (87.5%).

The majority of participants reported having health insurance (n = 66; 82.5%). Only 16.3% (n = 13 total in both groups) reported receiving routine or preventative care. However, nearly half of the study sample reported going to a clinic or doctor’s office most often for regular care thereby suggesting an opportunity to engage participants in a discussion about PrEP (Table 3). Additionally, more than half of participants (n = 50 total in both groups; 63%) reported receiving any medication during the past year (Table 3).

**Table 3:** Participant self-reported medical care/access.

	Control N = 39 (%)		Intervention N = 41 (%)		P-value
**Has Health Insurance**	32 (82.1)		34 (82.9)		P = 0.27
**Type of Health Insurance**					P = 0.90
*Private*	4 (10.3)		3 (7.3)		
*Medicaid/Medicare*	10 (25.6)		11 (26.8)		
*Unknown*	3 (7.7)		5 (12.2)		
**Receives preventative care**	7 (18.4)		6 (14.6)		P = 0.47
**Place you go most often for medical care**					P = 0.76
*Clinic/Health Center/ Doctors Office*	21 (53.8)		17 (42.5)		
*Hospital Emergency Room*	6 (15.4)		7 (17.5)		
*Hospital Outpatient Department*	0		1 (2.5)		
*Other/No Place/Don’t Know*	12 (30.8)		15 (37.5)		
**Last seen or talked to a medical provider about your health** *					P = 0.63
*Never*	5 (12.8)		3 (7.3)		
*Less than 6 months*	19 (48.7)		20 (48.8)		
*6 months– 1 year*	3 (7.7)		2 (4.9)		
*1–2 years*	2 (5.1)		5 (12.2)		
*2–5 years*	5 (12.8)		3 (7.3)		
*>5 years*	2 (5.1)		5 (7.3)		
**Complete Physical Exam in the past 2 years**	17 (43.6)		19 (46.3)		P = 0.58
**Pap Smear in past 2 years (female %)**	13 (76.5)		12 (66.7)		0.37
**Number of trips to ED for own health in past year** *	None	19 (48.7)	None	23 (56.1)	P = 0.60
	1	5 (12.8)	1	5 (12.2)	
	2–3	10 (25.6)	2–3	7 (17.1)	
	>3	5 (12.8)	>3	6 (14.6)	
**Number of clinic visits for own health in past year** *	None	15 (38.5)	None	16 (39)	P = 0.72
	1	5 (12.8)	1	5 (12.2)	
	2–3	10 (25.6)	2–3	10 (24.4)	
	>3	8 (20.5)	>3	4 (22)	
**Received any medication prescriptions in the past year**	25 (64.1)		25 (61.0)		P = 0.80

*Kruskal-Wallis Non-parametric Test was performed for ordinal data.

Chi-squared Test was performed for other variables in the table.

## Limitations

Our study is not without limitations. First, this is a pilot study with a relatively small number of participants (n = 80) so our findings may not be representative of criminal-legal involved individuals in other areas of the US or even in other part of Arkansas. Second, given the recruitment challenges associated with the COVID-19 pandemic, we had to change recruitment location from the county jail to local reentry centers. We noted that the demographics of the reentry centers differ somewhat from the demographics of individuals incarcerated in the county jail; individuals incarcerated in the jail are disproportionately African American and of a younger age. Additionally, we recognize the potential bias from self-report data for sensitive topics such as criminal history, substance use and HIV related risk behaviors. Our staff was able to develop rapport with participants through HIV education groups prior to participants enrolling in the study in order to minimize this potential bias. Finally, while questions related to substance use and healthcare utilization were intended to reference the timeframe prior to most recent incarceration, we cannot be certain participants responded in this manner as opposed to current use.

## Discussion

Individuals involved in the criminal-legal system face greater risk of adverse health outcomes, including HIV, due to social factors such as unstable housing and employment, lack of routine physical healthcare, and lack of treatment for mental illness and Substance Use Disorders (SUD). Individuals leaving incarceration in Arkansas are often reincarcerated within three years [51], creating additional barriers to a stable lifestyle that could promote preventative health practices or opportunities for SUD recovery. Disparities of HIV burden among race and geographic region compound these risk factors suggesting individuals cycling in and out of incarceration in Arkansas may have significant potential benefit from PrEP as an HIV prevention tool [19, 52].

This pilot randomized controlled trial was developed based on existing literature showing promising results for interventions linking individuals to HIV care [53–56] and treatment for opioid use disorder (OUD) [57] upon release from incarceration. Importantly, the literature suggests that ongoing support and individualized navigation may be important to sustaining positive health outcomes after initial linkage [58].

Baseline results demonstrate PrEP need and acceptance among participants, although barriers to PrEP access for these participants are great. We know that few Arkansans who are eligible for PrEP are actually prescribed PrEP [51] and social determinants of health, including housing and financial insecurity, common with frequent incarceration, present many competing priorities to accessing healthcare upon release [59].

Baseline results show a large potential benefit for PrEP uptake among this population. The navigation, support, and public health education provided by a CHW has the potential to address common barriers to accessing healthcare, including PrEP, among this population. While most participants reported little knowledge of PrEP before the study’s HIV class, participants overwhelmingly reported a willingness to take PrEP to help prevent HIV and to take a pill every day if it would prevent them from getting HIV. The majority of participants had health insurance at baseline, however, self-reported healthcare utilization was low. Outside of incarceration, many participants reported only seeing a medical professional when something was wrong, rather than engaging in routine preventative care where a PrEP prescription could be discussed.

The results of the completed RCT will highlight barriers to PrEP uptake and whether CHW navigation may help increase PrEP uptake among participants. The results of this pilot RCT will inform future PrEP linkage interventions designed for individuals ending their jail or prison sentence, particularly considering unique systems level challenges in the US south and within the public/private sphere of the reentry center setting where this intervention is initiated.

## Supporting information

S1 File (DOCX)

S1 Checklist CONSORT 2010 checklist of information to include when reporting a randomised trial*. (DOC)
