# Supplementary material for: Randomized controlled trial of a pilot PrEP linkage intervention for individuals leaving incarceration in a southern state: Design and baseline characteristics
Source: PLoS One. 2024 Dec 2;19(12):e0312667. doi: 10.1371/journal.pone.0312667 (PMC11611198; doi:10.1371/journal.pone.0312667)
Supplement: S1 File — (DOCX) [file pone.0312667.s001.docx]

**Study Title**: Linking High-Risk Jail Detainees to HIV pre-exposure Prophylaxis: PrEP-LINK

**Principal Investigator**: Nickolas Zaller, PhD

University of Arkansas for Medical Sciences

4301 W. Markham Street, Slot # 820

Little Rock, AR 72205

Telephone: *501.686-8366*

Email: *ndzaller@uams.edu*

**Sub-Investigator (s)**: Alex Marshall, PhD

University of Arkansas for Medical Sciences

4301 W. Markham Street, Slot #820

Little Rock, AR 72205

Telephone: *501-526-6623*

Email: *SMARSHALL@uams.edu*

**Study location**: University of Arkansas for Medical Sciences;

Pulaski County Detention Facility

2900 South Woodrow

Little Rock, AR 72204

Central Arkansas Community Corrections Center

4823 West 7^th^ Street

Little Rock, AR 72205

East Central Arkansas Community Correction Center

200 West Tyler Ave

West Memphis, AR 72301

Hidden Creek Opportunity Center

10905 W. Markham St.

Little Rock, AR 72211

Quality Living Center

3925 Asher Ave

Little Rock, AR 72204

Better Community Development, Inc.

4604 West 12^th^ Street

Little Rock, AR 72204

Recovery Centers of Arkansas

9219 Sibley Hole Road

Little Rock, AR 72209

And

6301 Father Tribou

Little Rock, AR 72205

# Table of Contents

[Table of Contents 2](#_Toc7953809)

[Background and Rationale 3](#_Toc7953810)

[Hypothesis *and/or* Specific Aims *or* Objectives 3](#_Toc7953811)

[Study Design and Procedures 5](#_Toc7953812)

[Study Population 11](#_Toc7953813)

[Inclusion Criteria: 12](#_Toc7953814)

[Exclusion Criteria 13](#_Toc7953815)

[Risks and Benefits 13](#_Toc7953816)

[Data Handling and Recordkeeping 13](#_Toc7953817)

[Data Analysis 14](#_Toc7953818)

[Ethical Considerations 16](#_Toc7953819)

[Dissemination of Data 17](#_Toc7953820)

[References 18](#_Toc7953821)

# Background and Rationale

Criminal justice (CJ) involved individuals have extremely high prevalence of risk factors associated with HIV infection including poverty, substance use, transactional sex, high numbers of sex partners, high prevalence of sexually transmitted infections and mental illness. African Americans (AA) are at disproportionate risk for both incarceration and HIV infections. **Arkansas incarcerates 1,020 people per 100,000 overall and 1,665 per 100,000 for African Americans, which substantially exceeds the 890 per 100,000 overall population in the United States.**^1-2^ Pulaski County, where this study will take place, has an HIV prevalence of nearly 500 HIV cases per 100,000 population.^3^

A growing body of evidence highlights the efficacy of a once daily medication, emtricitabine/tenofovir, also known as pre-exposure prophylaxis (PrEP), in reducing HIV acquisition.^4-8^ **However, structural and social barriers can undermine optimal PrEP uptake, adherence, and retention in care and must be addressed especially among those with recent CJ experience**. Recently incarcerated individuals often experience a lack of access to housing and employment, severance of social support, and discrimination—all of which are factors that can impede intervention efficacy and, therefore, must be incorporated into HIV prevention efforts. Thus, the primary goal of our proposed study is to develop a tailored PrEP intervention for CJ-involved populations, focusing on both individual and structural level factors, in order to reduce the risk for community based HIV acquisition. We will focus our study on the largest county jail in Arkansas and the largest community corrections office in the state. Jails, in contrast to prisons, are facilities where individuals are detained pre-sentencing for relatively short periods of time (days to months). Jail detainees are on the front lines of the HIV epidemic in the United States, yet barriers and facilitators to implementing PrEP – a highly effective intervention for reducing HIV risk – has not been studied in this critical population. There is also no research on PrEP uptake when offered as an intervention to individuals transitioning out of jail.

We have assembled an Interprofessional team of researchers with expertise in criminal justice, HIV and behavioral health. Our proposed study will: provide a novel exploration of facilitators and barriers to PrEP; allow us to target hard to reach populations, including men who have sex with men (MSM) and substance users (both of which are top funding priority groups for the National Institutes of Health) who experience intersecting markers of HIV risk; and inform regional, and possibly national, intervention approaches to combat the overlapping epidemic of HIV and incarceration.

# Specific Aims

The specific aims of the project associated with this protocol are:

**Aim 1:** **Perform a qualitative assessment of facilitators and barriers to PrEP uptake among high-risk jail detainees, systems and interactor levels.** To achieve this aim we will conduct qualitative interviews with three distinct groups of stakeholders:

1) Systems interviews (n=6);

2) Interactor interviews (n=8); and

3) Individual Key Informant interviews (n=20).

Within each of these strata, we aim to collect data representing diverse perspectives with respect to interest, knowledge, attitudes, and barriers (including substance use) to PrEP uptake. We will also explore acceptability and feasibility of an intervention to link individuals being released from the Pulaski County jail to PrEP services.

*For the purposes of this protocol, Aims 2 and 3 are identical in terms of participant recruitment, enrollment and all study procedures (including data collection and participant follow up). The primary objective of Aim 2 is to conduct a small pilot of the intervention study in order to make any necessary changes to any aspect of the study procedures. If any changes are made, those changes will be submitted to the IRB for further review and approval prior to initiating Aim 3. If no changes are made, this IRB protocol will serve as the protocol for both Aims 2 and 3.*

**Aim 2:** **Develop the PrEP-LINK intervention and perform an open label evaluation with high-risk HIV negative individuals being discharged from the Pulaski County Jail or recently discharged from a correctional setting and reporting to a local Community Corrections facility or Re-Entry Center, including: Central Arkansas Community Corrections Center, East Central Arkansas Community Corrections Center, Hidden Creek Opportunity Center, Quality Living Center, Better Community Development, Inc., and Recovery Centers of Arkansas, .**Directly informed from formative work (Aim 1), we will develop the PrEP-LINK intervention, which will include the use of a community health worker (CHW) and adherence counseling (based on ADAPT-ITT) approach to enhance PrEP care and overcome barriers to uptake in this population. We will then perform an open pilot with individuals recently released from a correctional facility

**Aim 3: Conduct a pilot RCT of the PrEP-LINK intervention among high-risk HIV negative individuals being released from the Pulaski County Jail or recruited within 30 days of release from a correctional facility.** Based on the formative work in Aim 1 and open pilot evaluation in Aim 2, participants will be randomized to either the PrEP-LINK intervention, or enhanced standard of care arm. Both arms will receive education about PrEP and referral to a community PrEP provider which exceeds the standards of care these individuals would otherwise receive.

While we will develop the CHW intervention as part of Aim 2, this will be informed by work we have already completed through Aim 1, which was approved under a separate IRB protocol (#207297). Once we have finalized the intervention content, we will conduct the Aim 2 open label evaluation in the exact same way as we will conduct the Aim 3 pilot RCT. To reiterate, the rationale of the open label evaluation proposed in Aim 2 is to ensure that the intervention is appropriate and does not need additional revision prior to conducting the Aim 3 RCT.

**Description of the Community Health Worker (CHW) Intervention (Aims 2 and 3)**: The intervention arm in both Aims 2 and 3 will receive additional support by a community health worker (CHW) who will assist with healthcare and social service navigation and will utilize adherence counseling techniques. The CHW we are employing is Ms. Timikia Jackson, who is a trained CHW with a strong knowledge base of both healthcare and social service systems in the greater Little Rock area. As per the model our intervention is based upon (the national Transitions Clinic Network [TCN], which has 14 sites across the country), Ms. Jackson has a history of incarceration. Therefore, she can serve as a true peer to individuals who are enrolled into the study. The national TCN sites employ formerly incarcerated CHWs because these individuals are often able to develop close relationships with their peers who have also been formerly incarcerated thereby building a strong foundation of trust among a population with high levels of distrust of the medical system. Ms. Jackson will be certified in HIV testing and risk reduction counseling. For participants randomized to the intervention group, Ms. Jackson will assist individuals with employment, housing (if needed), referrals to mental health or substance use treatment, keeping track of medical appointments and medication adherence. We anticipate that Ms. Jackson will be in contact with intervention arm participants at least once per week but the exact amount of contact for each participant will vary based on needs. Ms. Jackson will be available during regular business hours (Monday-Friday, 9 AM until 5 PM). If participants present to Ms. Jackson in crisis, she will refer them immediately to the Emergency Department, in the event of a medical or behavioral health emergency or the Pulaski County Regional Crisis Stabilization Center, which is operated by the UAMS Psychiatric Research Institute (PRI). Ms.Jackson will be directly supervised by PI Dr. Zaller through weekly supervision meetings and biweekly phone check-ins. Frequency of check-in may increase or decrease, as needed, during the course of the study period. Ms. Jackson is considered research staff and has been trained in human subjects through the UAMS CITI program.

Primary outcomes will include acceptability and feasibility of the intervention, as well as PrEP uptake. Secondary outcomes will include adherence and retention in PrEP-related medical care at three, six and 12 months. Results will inform a subsequent multi-site RCT to test efficacy of the intervention in improving PrEP uptake, adherence, and retention in care among transitioning out of jail.

***Between study aims 2 & 3 a total of 120 subjects will be consented with approximately 10 for aim 2 and 110 for aim 3.***

***NOTE: For this IRB application, we are only seeking approval for activities related to AIMs 2-3. AIM 1 was addressed in IRB #207297.***

# Study Design and Procedures

Human Subjects Involvement, Characteristics, and Design: Given the scope of inquiry and proposed project’s aims, criminal justice (CJ) involved individuals are the human subjects of this project. Our research team understands that CJ-involved individuals represent a vulnerable population, as the correctional setting may be perceived as a coercive environment. Therefore, all research must be conducted with rigorous ethical standards and with numerous safeguards in place to protect participants. A 2006 Institute of Medicine report on research involving prisoners called for the following requirements:

1. a risk-benefit approach, whereby the potential benefits of the research to the prisoners outweigh the potential risks;
2. research should be conducted with “collaborative responsibility,” referring to the idea that the design and implementation of the research should include the input of all relevant parties (including prisoners and correctional administrators);
3. voluntary informed consent must be obtained, and privacy maximized; and
4. research must be monitored throughout the course of the study in order to verify compliance with approved protocols and to detect and report adverse events. The research plan described in this application complies with all of these requirements. Below each aim and the relevant human participants’ involvement, characteristics and design are described.

There will be two groups that participants can be assigned to if they choose to participate in the study. Each person will be randomly assigned to one of these groups. One group will provide information about PrEP and HIV prevention and referral to PrEP clinics in the community. The second group will the same as the first group but will also provide access to a CHW who will assist them with getting to a PrEP clinic and also helping them with other needs they may have.

A member from the study team will review the informed consent documents with the prospective participants and ask them questions to make sure they are eligible to participate in this study (PrEP-LINK eligibility and demographic screening form). Their responses to the prescreen form will not be shared with anyone outside of the study and will not contain any identifying information. They will be kept completely private, and will be in a password protected, encrypted database on a secured server at UAMS that can only be accessed by designated study staff.

Before we collect this information, we ask that participants provide their name so that we can assign a 9-digit study-ID number to de-identify their responses. Names will be stored separately from responses in locked filing cabinet in a secured office and will never be connected. At the end of the study, the link between the name and the study ID will be permanently destroyed.

Random Assignment to Study Condition. Once qualified for the study, we will randomly assign them to one of the two study groups. Once assigned to a group, after the completion of the

| Table 4: Points of Contact for each Assessment for Aims 2 and 3 | | |
| --- | --- | --- |
| Type of Visit | Assessment | Location |
| Eligibility Screen | Education and Screening for initial PrEP indication | Pulaski County Jail, Central Arkansas Community Corrections Center, East Central Arkansas Community Corrections Center, Hidden Creek Opportunity Center, Quality Living Center, Better Community Development, Inc. |
| Consent, Enrollment | Baseline Research Assessment, HIV rapid testing to confirm HIV negative serostatus | CED Core community site |
| Presentation at PrEP Program for PrEP prescription dispensed | Baseline Research Assessment | Community based PrEP Provider |
| PrEP intervention sessions | Adherence assessment (self-report only), sexual risk ACASI, post-incarceration issues and HIV risk only | Community based PrEP provider or CED Core Community site |
| Short-term follow up assessment | Full assessment battery, including adherence assessment (self-report and clinical chart abstraction) | CED Core community site |
| Long-term follow up assessment | Full assessment battery, including adherence assessment (self-report and clinical chart abstraction) | CED Core community site |

baseline data collection measures the study research assistant will inform the community health worker and the participant to what group they were assigned to.

The participants will take place in the following activities over the course of 12 months:

- An HIV test using the FDA-approved OraQUICK ADVANCE® Rapid HIV Antibody Tests.
  - During the testing process the participant will receive:

(1) Pre-test counseling: A member of the research team, who has been trained by the Arkansas Department of Health to perform HIV counseling, will provide information on HIV and the meanings of a positive and negative test. Participants will have an opportunity to ask questions and will be offered prevention counseling.

(2) HIV testing: To test for HIV, a member of the research team will use the FDA approved OraQUICK ADVANCE® Rapid HIV Antibody Test.

(3) Post-test counseling: They will find out the results of their HIV test about 20 minutes after being tested. A member of the research team will go over the results of the test and engage in routine post-test counseling.

If tested positive for HIV, we will refer to the Arkansas Health Department for confirmatory testing and medical care. Study staff will also follow up to see if they received a confirmatory test. Additionally, any information collected up to that point will be destroyed and their participation in the study will be complete.

(4) PrEP counseling: Following screening and after the completion of the baseline assessment survey, participants will receive information about PrEP using materials developed by the Centers for Disease Control and Prevention (CDC). This includes watching a 2.5-minute video, and a brochure that lists local clinics where PrEP is available and how to make an appointment.

- - - If they decide to initiate PrEP, they will be prescribed PrEP medications by a physician in accordance with routine clinical care at their local PrEP clinic in the community.

Only participants who test negative for HIV on their rapid test will be eligible to participate in the study. Individuals in the jail with a preliminary positive HIV test result will be immediately referred to the jail medical division, operated by TurnKey, Inc. for confirmatory testing. Individuals tested in the community will be immediately referred to the Arkansas Department of Health for confirmatory testing. .

- A baseline assessment survey (SEE: PrEP-LINK Baseline Battery) to be used during all 3 data collection time points (baseline, 6-month and 12-month)
  - The baseline assessment survey will ask questions about education, job status, criminal justice history, substance use, sexual behaviors, and other behaviors which may put individuals at risk for HIV, in addition to questions on knowledge and interest in PrEP.
  - The baseline assessment survey includes the following measures and it to be used in both **Specific Aims 2 & 3**:

| **Measure Name** | **Citation** | **Baseline Battery Page Number** | **Notes** |
| --- | --- | --- | --- |
| PrEp-LINK Cover Sheet | Compiled for this study by the PIs | 1 | For internal tracking purposes |
| Demographics | Compiled for this study by the PIs | 2-5 |  |
| Criminal Justice Involvement History | Compiled for this study by the PIs | 5-6 |  |
| Health Insurance | Compiled for this study by the PIs | 6 |  |
| HIV Sex Risk Perception | Compiled for this study by the PIs | 7 |  |
| Income (ASI Lite) | McLellan, A.T., Luborsky, L., O’Brien, C.P. & Woody, G.E. (1980). An improved diagnostic instrument for substance abuse patients: The Addiction Severity Index. Journal of Nervous & Mental Diseases, 168, 26-33 | 8-9 |  |
| PrEP Knowledge (  PrEP Knowledge Scale, RADAR) | Parsons, J. T., Rendina, H. J., Lassiter, J. M., Whitfield, T. H., Starks, T. J., & Grov, C. (2017). Uptake of HIV pre-exposure prophylaxis (PrEP) in a national cohort of gay and bisexual men in the United States: the motivational PrEP cascade. *Journal of acquired immune deficiency syndromes (1999)*, *74*(3), 285. | 10 |  |
| PrEP Attitude | McLellan, A.T., Luborsky, L., O’Brien, C.P. & Woody, G.E. (1980). An improved diagnostic instrument for substance abuse patients: The Addiction Severity Index. Journal of Nervous & Mental Diseases, 168, 26-33 | 11 |  |
| PrEP Use | Morgan E, Moran K, Ryan DT, Mustanski B, Newcomb ME. Threefold increase in PrEP uptake over time with high adherence among young men who have sex with men in Chicago. AIDS Behav. 2018; 22(1):3637-3644. | 12-14 |  |
| PrEP Adherence | Chesney M.A., Ickovics J.R., Chambers D.B., Gifford, A.L., Neidig, J., Zwickl, B., & Wu, A.W.  (2000). Self-reported adherence to antiretroviral medications among participants in HIV clinical  trials: the AACTG adherence instruments. AIDS Care - Psychological and Socio-Medical Aspect  of AIDS/HIV, 12, 255-266.  Centers for Disease Control and Prevention. (2015). Medical Monitoring Project (MMP). Retrieved December 17th, 2017, from https://www.cdc.gov/hiv/pdf/statistics/systems/mmp/CDC-HIV-MMP-Questionnaire-2015-2017-EN.pdf  Morgan E, Moran K, Ryan DT, Mustanski B, Newcomb ME. Threefold increase in PrEP uptake over time with high adherence among young men who have sex with men in Chicago. AIDS Behav. 2018; 22(1):3637-3644. | 15-16 |  |
| HIV Attitudes (Perceived Risk of HIV Scale) | Napper, L.E., Fisher, D.G., & Reynolds, G.L.(2012). Development of the Perceived Risk of HIV Scale. *AIDS and Behavior*, *16*(4), 1075-1083. | 17-18 |  |
| HIV Risk – 30 Days (Texas Christian University HIV/Hepatitis Risk Assessment) | Simpson, D.D., Camacho, L.M., Vogtsberger, K.N., Williams, M.L., Stephens, R.C., Jones, A., & Watson, D.D. (1994). Reducing AIDS risks through community outreach interventions for drug injectors. *Psychology of Addictive Behaviors,* *8*, 86–101. | 19-20 |  |
| Alcohol Use (AUDIT) | Martin S, Glynn TR. Alcohol Use Disorder Identification Test. In: *The SAGE Encyclopedia of Alcohol: Social, Cultural, and Historical Perspectives*. ; 2015. doi:10.4135/9781483331096.n19 | 21-22 |  |
| Substance Use (ASI Lite) | McLellan, A.T., Luborsky, L., O’Brien, C.P. & Woody, G.E. (1980). An improved diagnostic instrument for substance abuse patients: The Addiction Severity Index. Journal of Nervous & Mental Diseases, 168, 26-33 | 23-28 |  |
| Drug Overdose | Compiled for this study by the PIs | 29-30 |  |
| Trauma (Traumatic Life Events Checklist) | Based on Life Events Checklist by: Gray, M., Litz, B., Hsu, J., & Lombardo, T. (2004). Psychometric properties of the Life Events Checklist. Assessment, 11, 330-341. doi: 10.1177/1073191104269954v  Weathers, F.W., Blake, D.D., Schnurr, P.P., Kaloupek, D.G., Marx, B.P., & Keane, T.M. (2013). The Life Events Checklist for DSM-5 (LEC-5). Instrument available from the National Center for PTSD at www.ptsd.va.gov | 31-32 |  |
| Depression (Patient Health Questionnaire – 9 item version for Depression Symptoms) | Kroenke, K., Spitzer, R. L., & Williams, J. B. (2001). The Phq‐9. *Journal of general internal medicine*, *16*(9), 606-613. | 33 |  |
| Discrimination (Major Experiences of Discrimination Scale) | Williams, D.R., Yu, Y., Jackson, J.S., & Anderson, N.B. (1997). Racial differences in physical and mental health: Socioeconomic status, stress, and discrimination. Journal of Health Psychology, 2(3), 335 -351 | 34-37 |  |
| Healthcare Access | National Health Interview Survey(CDC, 2018) | 38-42 |  |
| Health and Utilization | RAND-36 Item Health Survey, ASI Lite, Medial Mistrust Survey, Commonwealth survey, survey on Disparities in quality of Health Care 2001 | 43-44 |  |
| Medical Mistrust | Medical Mistrust Index v2.2 | 45-47 |  |
| Social Support (Multidimensional Scale of Perceived Social Support) | Zimet, G.D., Dahlem, N.W., Zimet, S.G. & Farley, G.K. (1988). The Multidimensional Scale of Perceived Social Support. Journal of Personality Assessment, 52, 30-41. | 48 |  |
| Civic Engagement | Compiled for this study by the PIs | 49 |  |

- HIPAA Release to UAMS
  - This will be completed at baseline to all medical records to be collected for the duration of their participation in the intervention regarding their PrEP initiation and adherence.

Follow-Up Assessments. Everyone will be asked to do follow-up assessments below:

**3 Month Locator Check in:**After 3 months, a member of the study team will contact the participant to see how they are doing (PrEP-LINK Locator Form).

**6 Month Assessment (Visit 2):**Participants will complete a survey that will take approximately one hour at a public location in Little Rock that is convenient for them (See: PrEP-LINK Baseline Battery). This may be UAMS, local PrEP clinic, a public library, or other private meeting room. This may also take place over the phone, Zoom, FaceTime, or other platform preferred by the participant to allow for social distancing during the Covid-19 pandemic.

**9 Month Locator Check in:**After 9 months, a member of the study team will contact them to see how they are doing (PrEP-LINK Locator Form).

**12 Month Assessment (Visit 3):**Participants will complete a survey that will take approximately one hour at a public location in Little Rock that is convenient for them (See: PrEP-LINK Baseline Battery). This may be UAMS, local PrEP clinic, a public library, or other private meeting room. This may also take place over the phone, Zoom, FaceTime, or other platform preferred by the participant to allow for social distancing during the Covid-19 pandemic.

Individuals who are eligible and willing to participate will be involved in the study for 12-months. All participants will complete 3 in-person interviews every 6 months and will receive $20 for their initial visit and $30 for the 6-month and 12-month follow up visits. Participants will also complete 2 brief phone check-ins between interviews, after which they will receive an additional $10 during their next in-person visit. Participants completing each interview and check-in will receive $100 for their time. During the first and last study visit, participants will need to complete a saliva/spit based HIV screening test.

Maintaining Contact. Because the study will take place over about 12 months after the participant’s enrollment date, it will be important for us to be able to keep in contact with them. During each visit, we will ask for information that will help us contact them in the community in order to schedule study related appointments. We will ask for many ways to try to reach the participant’s in case their phone number is disconnected or changed. We will contact them regularly to update your contact information.

# Study Population

Participants will be enrolled in the proposed PrEP-LINK intervention who are HIV-uninfected and detained in the Pulaski County jail or individuals who are within 30 days of having been released from a correctional setting. We will work with jail staff to identify individuals who are within 2 weeks of discharge and will conduct screenings among these individuals to determine study eligibility. We will also work with Central Arkansas Community Corrections and local re-entry centers to identify individuals who have been released from incarceration within 30 days. Because many types of HIV related risks are present in this population (e.g. MSM or PWID), we will recruit participants from the following groups: MSM, PWID and heterosexual non-substance users (more participants may be enrolled, as needed). For the final two categories, (PWID and heterosexuals), we will recruit equal numbers of men and women. The recruitment will be completed when 120 individuals have been consented to have 100 participants complete the protocol.

Inclusion criteria include: 1) being at least 18 years old; 2) having at least one self-reported HIV risk factor; and 3) currently incarcerated in the Pulaski County Jail or recently released (within 30 days) from a correctional facility and residing in Central Arkansas.. We will employ three primary methods of recruitment. The first will be educational groups in which a member of the study team will conduct weekly health related education sessions in the jail pertaining to HIV and prevention. Promotion of this health and wellness class will be conducted by fliers being posted in the jail facility outlining how detainees can enroll in the class provided by UAMS. We will indicate to both jail staff and to potential participants (during the beginning of each session) that these sessions are research related and strictly voluntary. We will also clearly state to jail staff to specifically tell jail detainees that participation in these session is voluntary and that the sessions are part of an ongoing research study. Among individuals who participate in these sessions, a member of the study team will approach potential participants individually, briefly explain the purpose and procedures for the intervention, and ask them if they wish to participate in the baseline interview expected to last about 60 to 90 minutes. The second method of recruitment will be referrals from medical staff at the jail. We will ask medical staff during their routine physical exam of all detainees to refer any individuals to a member of the study team who they feel may meet the study eligibility requirements. The third method of recruitment will be through local Community Corrections facilities and re-entry centers. Ms. Jackson will hold educational sessions at the recruitment sites (either in person or over Zoom for safety during the Covid-19 pandemic) where she will inform people about the study and conduct eligibility screeners with those who are interested, with the help of Research Assistants. All procedures will be reviewed by the UAMS institutional review board before the study begins. The PI has significant experience (nearly 15 years) with CJ-related research protocols.

## Inclusion Criteria:

1. Age greater than or equal to 18 at study enrollment
2. Able to understand and speak English and to provide written and verbal informed consent.
3. Able to provide reliable pieces of locator information (e.g. mailing address, phone number, email address, places frequently visited, etc.)
4. HIV negative confirmed through an on-site rapid HIV test;
5. Recently released from the Pulaski County Regional Detention Facility into the community and not into another detention facility or recently released (within 30 days) from a correctional facility and residing in Central Arkansas.
6. At substantial risk for HIV as determined by CDC/WHO PrEP guidelines, which includes engaging in any of the following behaviors in the last six months:

- Having condomless vaginal or anal sex with more than one partner,
- Having a sex partner with one or more HIV risk (e.g. PWID, a sex worker),
- Being diagnosed with a STI,
- Reporting history of sharing injection material/equipment, or
- Having a sexual partner who is HIV positive.

## Exclusion Criteria

Potential participants unable to provide informed consent, including people with severe mental illness requiring immediate treatment or with mental illness limiting their ability to participate (e.g., dementia), will be excluded.

# Risks and Benefits

While the risks related to human subjects’ participation in this study are minimal, we acknowledge that the possibility of harm to participants does exist. We recognize that participants will be asked to provide potentially sensitive information (including illicit substance use and sexual risk behaviors and criminal justice involvement) and therefore may experience discomfort, anxiety, or stress in responding to certain study questions. As such, as part of the informed consent procedure, participants will be advised that their answers will be confidential, and issues discussed will in no way impact their criminal justice status, nor ability to obtain or be referred to medical care or social services. At the start of the interview, participants will be informed that they can skip and refuse to answer any questions that they may find uncomfortable or embarrassing.

The potential benefits to participants include reduction in risk of HIV acquisition with use of pre-exposure prophylaxis. If effective, the proposed intervention is likely to substantially improve adherence to PrEP and thereby increase the efficacy of the medication in preventing HIV seroconversion, particularly among individuals with criminal justice experience. Reduction in the incidence of HIV infection among individuals enrolled is also likely to lead to decreased transmission in the community as a whole. Study participants will be compensated for their participation. Some may feel empowered by being involved in research that may impact correctional and community health policy and practice and may enjoy the interaction with members of the study team.

# Data Handling and Recordkeeping

Protecting Confidentiality: All members of the research team will treat participant data with the strictest attention to confidentiality. Data will be collected from participants in settings in the community and in correctional facilities in such a way that neither privacy nor confidentiality is compromised. All records and data will be safeguarded in accordance with the policies of the participating IRB. Questionnaires will not include participants’ name or any other identifying information. Data for this phase of the study will be brief eligibility screens study (see Appendix PrEP-LINK eligibility and demographic screening form) and the baseline surveying tool (see Appendix PrEP-LINK Baseline Battery). Only members of the study team (PI Dr. Zaller, co-Is Dr. Marshall and the study RAs, Heather Horton and Brooklyn Tody) will have access to the data. At the conclusion of the study, the data will be stored electronically until analyses are completed. Electronic Data will be kept for the requisite 7 years, as per UAMS administrative guidelines.

Before we collect any information, we ask that participants provide their name so that we can assign a 9-digit study-ID number to de-identify their responses. Names will be stored separately from responses in locked filing cabinet in a secured office and will never be connected. At the end of the study, the link between the name and the study ID will be permanently destroyed. The participant ID link file will be kept separately in a password-protected folder/document on a secure UAMS server. All locator forms will be stored separately from the responses and destroyed at the completion of data collection.

# Data Analysis

**Measures.** The primary intervention measures, including common measure across both studies are provided above in the description of Aim 2. As part of Aim 3, data will be collected at all follow-up time points (3, 6 and 12 months) by the study RA. Additional measures are provided below.

**Additional Secondary Measures for Aim 3**

**1)** **PrEP adherence.** Self-reported PrEP adherence will supplement clinical chart abstraction. An assessment of the optimal period for self-reported antiretroviral adherence (using electronic monitoring data as the standard for predictive validity) found that longer time periods (e.g., 30 days versus 4 days) were more accurate for assessing adherence than shorter time periods.^51^ Therefore, we will use adherence over a 30-day period as self-reported adherence outcome.

**2) HIV risk behaviors/behavioral adjustment.** Participants’ HIV-transmission risk behaviors will be assessed with the TCU HIV/AIDS Risk Assessment, which is designed specifically for CJ-involved individuals,^52^ at all three data collection time points. We will collect more detailed data on sexual behaviors, including number of sexual partners, sexual activities, and condom use based on EXPLORE study measures. These measures are valid and reliable among high-risk men.^53^

**3) Substance use.** Participants’ alcohol use will be measured with the Alcohol Use Disorders Identification Test - Consumption (AUDIT-C) and the Addiction Severity Index - Lite (ASI-Lite), both of which have been validated for use with diverse populations (e.g., African American, Hispanic/Latino, and White).^56^

**Data analysis.** Data analysis will focus on the feasibility, acceptability, and preliminary efficacy of the PrEP-LINK pilot intervention.

*The feasibility* of the intervention will be determined by the proportion of participants who are screened, enrolled, and subsequently complete the PrEP-LINK intervention. To determine the feasibility of conducting an RCT, we will track the number of jail-involved individuals who express interest in the study and who are determined eligible for participation. Among these, we will calculate the percentages of those who reported being interested who were actually eligible; the number eligible who were successfully recruited and consented; randomized; and assessed at baseline, 3, 6, and 12 months (enrollment and completion of study related activities for >80% will indicate study feasibility). We will relay in individual detainees self-reporting in interest in participating in the study in the following ways. Firstly, the study CHW will be conducting weekly educational sessions. At the end of these sessions, individuals can disclose through an evaluation form whether or not they are interested in the study and if they are, a member of the study team will set up a follow up appointment to assess eligibility. The second way is direct referrals from the medical staff who may, with the permission of the detainee, make a referral to the study for eligibility assessment. The third method of recruitment will be through local Community Corrections facilities and re-entry centers. The study CHW will hold educational sessions at the recruitment sites (either in person or over Zoom for safety during the Covid-19 pandemic) where she will inform people about the study and conduct eligibility screeners with those who are interested, with the help of the research team.

*The acceptability* of the intervention will be determined with descriptive statistics of the participants’ acceptability ratings, including whether they think the intervention is acceptable. Acceptability is define as > 80% of participants expressing high satisfaction with the intervention based on the Client Satisfaction Questionnaire - Revised. We will also review data collected by the study staff about time spent on the intervention and the ease of following the research and intervention protocols.

*The preliminary efficacy of the intervention* will be determined by assessing the primary outcome of PrEP uptake by looking at the medical charts of the participants. A secondary outcome will be PrEP adherence defined by participants who were adherent to the medication determined by self-report at 3, 6, and 12 months. Following intent-to-treat (ITT) principles, all randomized participants will be included in data analyses. Standard bivariate analytic techniques, such as Spearman rank-order correlations, odds ratios, and Fisher's exact tests, will be used to assess preliminary associations between the intervention and each measure of PrEP uptake and to identify factors associated with the outcomes. Generalized linear regression models will be informed by these bivariate analyses.

In addition, we will *test the randomization process* by comparing the baseline demographic and confounding variables of the participants in each arm with Fisher's exact test for dichotomous variables, chi-square tests for nominal variables, *t*-tests for normally distributed continuous variables, and Wilcoxon signed-rank tests for non-normally distributed continuous variables, with statistical significance determined at alpha < 0.05. Any variable for which randomization does not result in equal proportions in each arm will be incorporated into the multiple-regression models as a potential confounding variable. Attrition effects will be evaluated by testing whether systematic differences exist between those participants who complete the research versus those who drop out. Thus, we can determine any potential bias introduced by attrition from the study.

*The primary analysis* will compare uptake of PrEP by looking at the medical charts of the participants and self-reported behaviors of PrEP usage at the 3, 6, and 12 month visits between the study arms. Changes in self-reported adherence (verification with clinical chart abstraction, including lab tests for serum levels of medications), sexual behavior adjustment, post-incarceration contextual variables, and substance use will also be examined. Moreover, group differences in PrEP uptake, time-to-uptake, and number of PrEP clinic appointments kept will be compared. All analyses will use two-tailed tests of significance, with significance at alpha = 0.05. We will use generalized linear models with properly chosen link functions to analyze longitudinal data for each study aim. These models will be estimated with generalized estimating equations that use robust standard error estimates^57,58^; they provide an extension of regression analysis to the case of correlated or repeated observations and allow for the inclusion of categorical and count-dependent variables and for appropriate modeling of covariance structures when observations are correlated across time.

**Power Calculations.** Because this is a pilot study, the primary emphasis will be on the acceptability and feasibility of the two arms; hence, power is limited to determine efficacy of the intervention. We used GPower^59^ software to estimate the difference in proportions between the two groups, with respect to PrEP-LINKage, that could be detected with 80% power. Given 40 participants per group (total *n* = 80), one-tailed alpha = .05, and a proportion of 0.5 for the control group, we will have 80% power to detect a 0.26 difference in proportions between groups.

# Ethical Considerations

This study will be conducted in accordance with all applicable government regulations and University of Arkansas for Medical Sciences research policies and procedures.  This protocol and any amendments will be submitted and approved by the UAMS Institutional Review Board (IRB) to conduct the study.

The informed consent process will include a detailed verbal description of the study by a study staff member. The study staff will ask participants a series of questions to ensure that the information contained in the consent form is clearly understood by the participants prior to consenting to participation. Study staff will emphasize that participation is voluntary. Potential subjects will participate in an item-by-item reading of the consent from with the study staff person.

The consent process will take place in a quiet and private room (either in a small conference room in the jail, Community Corrections and re-entry centers, or in a community location), and participants may take as much time as needed to make a decision about their participation. The consent process may also take place over the phone, Zoom, FaceTime, or other platform preferred by the participant to allow for social distancing during the Covid-19 pandemic. Participation privacy will be maintained and questions regarding participation will be answered. No coercion or undue influence will be used in the consent process.

In addition, we have a **Certificate of Confidentiality** from the National Institutes of Health. The purpose of this certificate is to protect the identity of research subjects participating in studies that collect sensitive information. Because this application proposes to collect detailed HIV sexual and substance use behavior among those with criminal justice system involvement, a Certificate of Confidentiality is necessary.

# Dissemination of Data

Results of this study may be used for presentations, posters, or publications. The publications will not contain any identifiable information that could be linked to a participant.

# References

1. Mauer M. Addressing racial disparities in incarceration. *Prison J*. 2011;91(3_suppl):87S–101S.

2. Nellis A. The color of justice: Racial and ethnic disparity in state prisons. *Sentencing Proj Available Httpwww Sentencingproject Orgwp-Contentuploads201606-Color--Justice-Racial--Ethn-Disparity--State-Prisons Pdf Accessed 22 Aug 2016*. 2016.

3. *HIV Survelliance Report Arkansas, 2015*. Arkansas Department of Health; 2015. http://www.healthy.arkansas.gov/programsServices/healthStatistics/Documents/STDSurveillance/HIVAIDSAnnualReport.pdf;2015. Accessed May 2, 2017.

4. Grant RM, Lama JR, Anderson PL, et al. Preexposure chemoprophylaxis for HIV prevention in men who have sex with men. *N Engl J Med*. 2010;2010(363):2587–2599.

5. Thigpen MC, Kebaabetswe PM, Paxton LA, et al. Antiretroviral preexposure prophylaxis for heterosexual HIV transmission in Botswana. *N Engl J Med*. 2012;367(5):423–434.

6. Baeten JM, Donnell D, Ndase P, et al. Antiretroviral prophylaxis for HIV prevention in heterosexual men and women. *N Engl J Med*. 2012;367(5):399–410.

7. Van Damme L, Corneli A, Ahmed K, et al. Preexposure prophylaxis for HIV infection among African women. *N Engl J Med*. 2012;367(5):411–422.

8. Volk JE, Marcus JL, Phengrasamy T, et al. No new HIV infections with increasing use of HIV preexposure prophylaxis in a clinical practice setting. *Clin Infect Dis*. 2015;61(10):1601–1603.

9. Guest G, MacQueen KM, Namey EE. Validity and reliability (credibility and dependability) in qualitative research and data analysis. *Appl Themat Anal Lond Sage Publ*. 2012:79–106.

10. Gale NK, Heath G, Cameron E, Rashid S, Redwood S. Using the framework method for the analysis of qualitative data in multi-disciplinary health research. *BMC Med Res Methodol*. 2013;13(1):117.

11. Carey JW, Morgan M, Oxtoby MJ. Intercoder agreement in analysis of responses to open-ended interview questions: Examples from tuberculosis research. *Cult Anthropol Methods*. 1996;8(3):1–5.
